# Management practices and continuing education in the treatment of canine idiopathic epilepsy among Indian primary care veterinarians

**DOI:** 10.3389/fvets.2026.1868929

**Published:** 2026-07-21

**Authors:** A. T. Srinath, K. R. Muñana, P. Deshmukh, R. Caddiell, M. E. Gruen, M. J. Lewis

**Affiliations:** 1 Department of Clinical Sciences, College of Veterinary Medicine, North Carolina State University, Raleigh, NC, United States; 2 Acumed Veterinary Specialty, Mumbai, India

**Keywords:** dog, levetiracetam, phenobarbital, seizures, survey

## Abstract

**Background:**

In India and countries without veterinary neurologists, dogs with idiopathic epilepsy (IE) are managed by primary care veterinarians. Data are lacking on their practice patterns.

**Objectives:**

Describe management of canine IE among Indian primary care veterinarians and explore the impact of access to educational materials about epilepsy.

**Methods:**

Prospective, two-phased, survey-based study of Indian veterinarians focused on treatment and monitoring of canine IE and perceived confidence in management recommendations (1/not comfortable to 5/very comfortable). Participants who agreed to Phase II received concise guidelines about IE with a follow-up survey distributed after 6 months. Descriptive statistics summarized management trends and compared confidence scores between surveys.

**Results:**

187 veterinarians participated in Phase I and of these, 36 participated in Phase II. Phenobarbital was the most common anti-seizure drug prescribed, chosen mostly due to efficacy. Most respondents (118/187; 63%) recommended routine lab work and (84/187; 45%) recommended measuring phenobarbital or potassium bromide blood concentration every 6–12 months; (61/187; 33%) reported not measuring blood concentrations routinely. Long-term management of IE and adverse effects of medications were rated as most challenging. Nearly half of Phase II respondents (17/36; 47%) ranked the educational materials as extremely beneficial. Mean self-reported comfort level was 3.3 (SD 0.97) and 3.69 (SD 0.89) for Phase I and II, respectively; most (20/36; 56%) reported no change between surveys.

**Conclusion:**

Challenges for Indian veterinarians in the chronic management of canine IE related to anti-seizure drug monitoring and adverse effects. Exploratory Phase II findings identified positive reception to educational support from specialists.

## 1 Introduction

Idiopathic Epilepsy (IE) is the most common neurologic disease in dogs worldwide with a reported prevalence of 0.62–0.82% in the general population (1–3). According to the International Veterinary Epilepsy Task Force, diagnosis of IE in dogs ideally includes confirmation of a normal brain on magnetic resonance imaging and cerebrospinal fluid analysis (4). However, a presumptive diagnosis based on a lower tier of evidence (e.g., typical age of onset, normal inter-ictal neurologic exam, exclusion of toxic and metabolic causes of seizures) is common in clinical settings, especially regions where access to specialty veterinary medicine is limited (4). Several reviews, consensus statements and meta-analyses compile available evidence and provide guidelines for the management of IE in dogs including the use of anti-seizure drugs (ASDs) and subsequent drug monitoring recommendations (5–7). Prior studies focusing on management practices from the United States (US), Netherlands, United Kingdom (UK), Australia and Japan provide region-specific insight (8–13). For example, phenobarbital is the most commonly utilized medication except in Japan where zonisamide is used overwhelmingly more frequently than in other countries (8–13). Similarly, there is variability regarding measuring serum concentrations of ASDs and performing routine bloodwork in dogs with IE (8, 9, 12). However, prior studies are from high income, developed countries (8–13).

The pet dog population in India reached 33.6 million in 2023, up from 12.6 million in 2014 (14, 15), which translates to potentially more than 200,000 dogs living with IE in the country. There are more than 80,000 veterinarians registered with the regulatory agency Veterinary Council of India (16). While India has a large number of veterinarians and veterinary colleges, advanced training programs focused on specialty care are lacking and well-equipped veterinary hospitals are still rare, especially outside major cities.

With the dearth of veterinary neurology specialists in India, canine IE is primarily managed by primary care veterinarians. It has been demonstrated generally that primary care practitioners in developed countries commonly obtain evidence to inform their clinical practice from journals, closely followed by electronic resources (e.g., Veterinary Information Network), though continuing educational support from veterinary specialists is also valued (9, 17, 18). However, the management practices of Indian veterinarians, what resources they are utilizing and the extent to which they are conforming to published guidelines on canine IE are currently unknown. Given the already sizable and growing number of epileptic pet dogs in India, gathering this information has important implications for ensuring appropriate veterinary care for this common, chronic condition in India and potentially other developing countries.

As with many chronic illnesses, cost of medications to manage IE can exert financial strain on pet owners. The median monthly expenditure for ASD medications in the United States is $51–75 (19), which has likely increased since initially reported. In human epilepsy management, high cost of treatment is a major reason for decreased adherence in resource poor countries, and substantial cost disparities among different brands of ASDs in India have been suggested to affect prescriber preferences, patient compliance and disease outcomes (20, 21). Factors, including cost, influencing practice patterns have not been described for Indian veterinarians managing epileptic dogs.

The primary aim of this study was to describe current management practices for canine IE among Indian primary care veterinarians including rationale for decision-making and perceived confidence in their clinical recommendations. A secondary aim was to preliminarily explore the impact of study-specific educational materials about canine IE on clinical practice, practitioner confidence and perceived challenges in implementation of guidelines. We hypothesized that treatment decisions for epileptic dogs were primarily based on cost and convenience and that access to simplified treatment and monitoring guidelines would improve practitioner confidence in their management of IE.

## 2 Materials and methods

### 2.1 Study design and study population

This was a prospective, 2 phased, survey-based study. The study was approved for exempt status by the Institutional Review Board and all respondents provided informed consent. The inclusion criteria for participation were being licensed to practice veterinary medicine in India, working in primary care veterinary practice in India, and having treated at least two dogs with IE in the previous 12 months. Respondents were excluded if they possessed an advanced degree or board certification or had undergone structured training in veterinary neurology. Respondents were recruited via email, social media platforms and advertisements at veterinary conferences in India. Only one response per participant was allowed.

Phase I consisted of an online questionnaire which was open to responses between January 2025 to March 2025. Respondents from Phase I who consented to participate in Phase II received educational materials created for this study that they were instructed to view at their discretion. After a period of 6 months, a follow-up online survey was distributed between July 2025 to September 2025. The 6-month timeframe between Phases I and II was selected to ensure respondents would likely manage one or more dogs with IE prior to completing the second survey. Phase II was designed as an uncontrolled, exploratory pilot study to explore the feasibility, reception and initial impact of educational materials on self-reported clinical confidence and management practices; therefore, a formal power calculation was not performed. Target sample sizes were informed by prior survey-based studies on canine IE, with an anticipated response of >200 respondents for Phase I and >50 respondents for Phase II.

### 2.2 Questionnaires

Questionnaires were adapted from a previous survey-based epilepsy study (8) and developed by a neurology resident under the guidance of board-certified neurologists, co-investigators with extensive survey methodology experience, and with additional regional insight from an Indian veterinary practitioner with special interest in the management of neurological conditions. Data were collected using online surveys created in English and administered via Qualtrics (Provo, UT, https://www.qualtrics.com/). Fraudulent responses by unqualified individuals or artificial bots were eliminated using the reCAPTCHA score in Qualtrics. Additional manual screening was used to check for and remove duplicate responses from the same or extremely similar email address and by reviewing initial eligibility questions, excluding responses with inconsistencies, mostly incomplete or unintelligible answers or where study eligibility could not be confirmed.

Question format was mostly multiple-choice including a proportion where respondents could select multiple answers. Where applicable, questions included an option for “Other” with a free text field to provide information not included in the available choices. Other question formats included a Likert-type scale to assess respondents’ attitude towards specific subjects, ranking questions (e.g., most to least common) and free response questions to provide specific, open-ended information. Content focused primarily on chronic management of IE and not on emergency management of seizures.

The Phase I survey documented demographic and background information including practice size and location, participants’ veterinary experience and caseload of epileptic dogs (Supplementary material 1). The questions were organized into sections relating to diagnosis of IE, initiation of therapy, selection of ASDs, monitoring and adjustment of ASDs and determination of treatment efficacy. Participants were also asked about their comfort with managing IE cases, challenges faced during management and their pursuit and perceived value of supportive information on IE. The Phase II survey was similar in structure and content to the first, with additional questions focusing on the educational materials including feasibility and frequency of use, perceived impact on decision-making, current confidence in the management of canine IE and any additional feedback on the educational materials (Supplementary material 2).

### 2.3 Educational materials

Using published, evidence-based guidelines on canine IE (4–7), combined with clinical expertise in the field, educational materials were developed by a neurology resident under the guidance of board-certified neurologists. Information was provided in English and was tailored to reflect regionally available ASDs and resources. Topics included guidelines and simplified treatment and monitoring algorithms on when and how to initiate and adjust ASDs, when and how to monitor dogs on ASDs including identifying adverse effects, and how to determine treatment success (Supplementary material 3).

### 2.4 Data analysis

Descriptive statistics were used to summarize and compare survey responses between Phase I and Phase II. Categorical variables were reported as proportions (*n*/*N*; %), while continuous variables were assessed for normality and presented as mean ± standard deviation (SD) or median (range) as appropriate. Comparisons between groups and phases were descriptive given the preliminary, exploratory nature of the study.

## 3 Results

### 3.1 Phase I

#### 3.1.1 Demographic and practice details

One hundred and eighty-seven veterinarians working in primary care in India completed the Phase I survey. One hundred and twenty-one (121/187; 65%) respondents were male, 64/187 (34%) were female, and 2 respondents preferred not to say (2/187; 1%). Median participant age was 28 years (range 23–66). Distribution of respondents by clinic and clinician factors is outlined in Table 1.

**Table 1:** Breakdown of Phase I and II survey respondents by clinic and clinician factors.

Category	Phase I (*N* = 187)	Phase II (*N* = 36)	Phase II non-responders (*N* = 151)
Practice type			
Urban	156 (83%)	32 (89%)	124 (82%)
Rural	31 (17%)	4 (11%)	27 (18%)
Clinic size			
Single vet	76 (41%)	20 (56%)	56 (37%)
2–5 vets	59 (31%)	7 (19%)	52 (34%)
>5 vets	52 (28%)	9(25%)	43(29%)
Experience in clinical practice			
<5 years	108 (58%)	23 (64%)	85 (56%)
>5 years	79 (42%)	13 (36%)	66 (44%)
Epilepsy caseload			
<5 cases/year	119 (64%)	23 (64%)	96 (64%)
>5 cases/year	68 (36%)	13 (36%)	55 (36%)

#### 3.1.2 Diagnosis

In the 12 months prior to undertaking the survey, most respondents treated fewer than 5 new cases of canine IE (119/187; 64%). Diagnostic criteria choices overall and stratified by clinic and clinician factors are depicted in Table 2; most selected more than one option. The most chosen criteria was a history of more than one generalized seizure and only a small number of respondents indicated utilizing magnetic resonance imaging and cerebrospinal fluid analysis. When performed, serum ammonia concentration elevation most commonly prompted pre- and postprandial bile acid testing to confirm suspicion of hepatic dysfunction as the cause of seizures (97/187; 53%). Alternatively, similar numbers of respondents considered hyperammonemia as the cause of seizures if elevated by more than twice the reference range (64/187; 34%) or with any elevation in serum ammonia (59/187; 32%).

**Table 2:** Frequency of criteria used by respondents for the diagnosis of canine idiopathic epilepsy (IE), stratified by practice and clinician characteristics.

Respondent category		Age of onset 6mo-6 yr	Normal lab work	Normal hepatic function	Normal brain MRI, CSF	Normal interictal exam	History >1 generalized seizure
Overall (*N* = 187)		132 (71%)	128 (68%)	105 (57%)	38 (21%)	141 (75%)	153 (82%)
Practice type	Rural (*N* = 31)	21 (68%)	11 (35%)	7 (23%)	2 (6%)	15 (48%)	20 (65%)
	Urban (*N* = 156)	111 (71%)	117 (75%)	98 (63%)	36 (23%)	126 (81%)	133 (85%)
Practice size	Single vet (*N* = 76)	52 (68%)	41 (54%)	26 (34%)	6 (8%)	48 (63%)	56 (74%)
	2–5 vets (*N* = 59)	39 (66%)	44 (75%)	39 (66%)	12 (20%)	50 (85%)	50 (85%)
	>5 vets (*N* = 52)	41 (79%)	43 (83%)	40 (77%)	20 (38%)	43 (83%)	47 (90%)
Experience	<5 yr. (*N* = 108)	72 (67%)	62 (57%)	51 (47%)	15 (14%)	73 (68%)	85 (79%)
	>5 yr. (*N* = 79)	60 (76%)	66 (84%)	54 (68%)	23 (29%)	68 (86%)	68 (86%)
Caseload	<5 cases/yr. (*N* = 119)	95 (80%)	87 (73%)	66 (55%)	20 (17%)	98 (82%)	106 (89%)
	>5 cases/yr. (*N* = 68)	37 (54%)	41 (60%)	39 (57%)	18 (26%)	43 (63%)	47 (69%)

Respondents could select more than one answer. mo, month; yr., year; MRI, magnetic resonance imaging; CSF, cerebrospinal fluid analysis.

#### 3.1.3 Treatment

**Figure 1:**
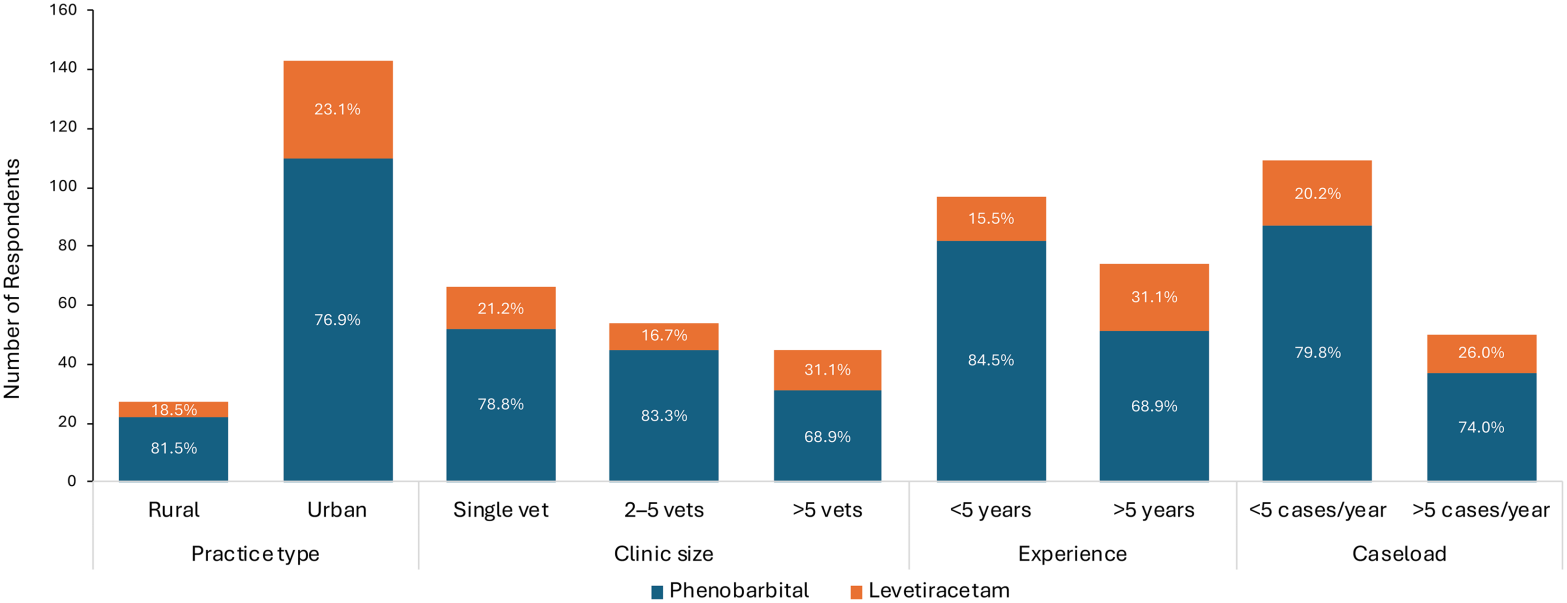
Stacked column chart illustrating respondents’ selection of phenobarbital or levetiracetam as the first-line anti-seizure drug (ASD) for canine idiopathic epilepsy (IE) stratified by practice type, practice size, experience, and caseload. Values are displayed as percentages within each subgroup.

The most common reasons chosen for starting an ASD were: 2 or more seizures occurring within 24 h (119/187; 64%), multiple seizures in the preceding 3–6 months (118/187; 63%), and a seizure lasting more than 5 min (91/187; 49%). A minority of respondents indicated prescribing ASDs after the first seizure (37/187; 20%). Phenobarbital was the most commonly prescribed first-line maintenance ASD (133/187; 71%), followed by levetiracetam (38/187; 21%), with potassium bromide (10/187; 5%) and zonisamide (6/187; 3%) prescribed uncommonly. The proportion of respondents choosing phenobarbital or levetiracetam stratified by demographic factors are outlined in Figure 1.

The most common reason for choosing the first ASD was efficacy in nearly half of respondents, followed by availability in the area in just over a quarter (Table 3). The reasons selected differed in frequency between practice type and which medication was selected. Among the dogs they managed for IE, most respondents (150/187; 80%) reported fewer than 50% were treated with two or more maintenance ASDs. The most common ASDs prescribed as add-on medication were phenobarbital or levetiracetam, at similar frequency, with potassium bromide chosen infrequently. The reasons for choosing the add-on ASD were based on efficacy in the majority (Table 3). Other treatments recommended by a minority of respondents for IE included cannabidiol supplementation, laser therapy/physical rehabilitation, herbal supplementation, dietary supplementation with medium chain triglycerides and least commonly, acupuncture.

**Table 3:** Frequency of reasons selected for choosing the first and add-on ASDs.

First ASD reason		Efficacy	Availability in area	Side effect profile	Cost	Ease of administration	Other reasons
Overall (*N* = 187*)		89 (48%)	53 (28%)	25 (13%)	7 (4%)	6 (3%)	5 (3%)
Practice type	Rural (*N* = 31)	8 (26%)	18 (58%)	2 (6%)	1 (3%)	1 (3%)	1 (3%)
	Urban (*N* = 156)	81 (52%)	35 (22%)	23 (15%)	6 (4%)	5 (3%)	4 (3%)
Medication	Phenobarbital (*N* = 133)	65 (49%)	40 (30%)	11 (8%)	6 (5%)	6 (5%)	5 (3%)
	Levetiracetam (*N* = 38)	12 (32%)	8 (21%)	17 (45%)	1 (3%)	0 (0%)	0 (0%)
Add-on ASD reason		Efficacy	Availability	Side effect profile	Cost	Different Route of metabolism	Other Reasons
Overall (*N* = 187**)		128 (68%)	78 (42%)	81 (43%)	49 (26%)	81 (43%)	81 (43%)

*2 respondents did not answer this question. **Respondents could select more than one answer.

#### 3.1.4 Monitoring

When prescribing ASDs, routine bloodwork was recommended to be performed regularly by most respondents; this was more common among urban compared to rural practitioners (Table 4). When prescribing phenobarbital or potassium bromide, respondents most commonly reported performing ASD concentration regularly (Table 4). Not measuring blood concentration of ASDs was more commonly chosen by respondents from clinics with 5 or fewer veterinarians in comparison to practices with more than 5 veterinarians. The most cited reasons for not performing ASD blood concentrations were pet owner financial restrictions (130/187; 70%) and lack of access to these lab tests (72/187; 39%).

**Table 4:** Frequency of reasons selected to perform routine bloodwork and ASD levels when phenobarbital or potassium bromide are prescribed.

Routine bloodwork monitoring		Performed every 6–12 months	If adverse effects	Poor seizure control	Not routinely performed	After initiation of ASD	After dose change
Overall (*N* = 187)		118 (63%)	46 (25%)	46 (25%)	11 (6%)	NA	NA
Practice type	Rural (*N* = 31)	13 (42%)	14 (45%)	8 (26%)	4 (13%)	NA	NA
	Urban (*N* = 156)	105 (67%)	32 (30%)	38(21%)	7(4%)	NA	NA
ASD levels							
Overall (*N* = 187)		84 (45%)	42 (22%)	NA	61 (33%)	46 (25%)	52 (28%)
Clinic size	Single vet (*N* = 76)	21 (28%)	22 (29%)	NA	34 (45%)	10 (13%)	15 (20%)
	2–5 vets (*N* = 59)	29 (49%)	14 (24%)	NA	21 (36%)	17 (29%)	18 (31%)
	>5 vets (*N* = 52)	34 (65%)	6 (12%)	NA	6 (12%)	19 (37%)	19 (37%)

Respondents could select more than one answer.

When faced with common side effects of ASDs (e.g., sedation, lethargy, ataxia), common responses included gradually tapering the dose and eventually discontinuing the ASD (122/187; 65%), performing bloodwork and ASD levels (91/187; 49%) or, contrastingly, continuing the ASD and monitoring for resolution of side effects (79/187; 42%). Regarding changes on bloodwork that respondents interpreted as indicating phenobarbital-induced hepatotoxicity, progressive alkaline phosphatase (ALP) elevation more than 2 times the high end of the reference interval (84/187; 45%) and any ALP elevation (82/187; 44%) were most common. Other lab work changes utilized included alanine aminotransferase (ALT) elevation greater than ALP (73/187; 39%), a decrease in liver function parameters (64/187; 34%) and/or any ALT elevation (22/187; 12%). Additional diagnostics recommended for suspected phenobarbital-induced hepatotoxicity included phenobarbital serum concentration (112/187; 60%), serum ammonia concentration (106/187; 57%), pre- and post-prandial bile acids (95/187, 51%), and/or abdominal ultrasound or computed tomography (74/187; 40%).

Recommendations chosen for treating phenobarbital-induced hepatotoxicity included gradually tapering phenobarbital dose for eventual discontinuation (73/187; 39%), decreasing the phenobarbital dose and monitoring bloodwork and phenobarbital blood concentration for improvement (60/187; 32%), initiating hepatoprotective treatment (e.g., Denamarin, Silybin) while continuing phenobarbital dose unchanged (43/187; 23%), or discontinuing phenobarbital without tapering (5/187; 3%). The most common recommendations for treating idiosyncratic adverse effects of phenobarbital (e.g., pancytopenia, necrolytic dermatitis, lymphadenopathy) were evenly distributed between gradually tapering phenobarbital dose for eventual discontinuation (82/187; 44%) or decreasing phenobarbital dose and monitoring for improvement (80/187; 43%). Regarding management for bromide toxicity, most respondents recommended hospitalizing on IV fluids (113/187; 60%).

#### 3.1.5 Treatment success and medication adjustments

An equal number of respondents chose the main goal when treating dogs with IE as either achieving complete seizure freedom (80/187; 43%) or reducing seizure frequency and severity but not complete freedom (79/187; 42%). Minimizing side effects associated with ASDs (17/187; 9%) and minimizing pet owners’ financial burden associated with seizure management (4/187; 2%) were uncommonly selected. Seven respondents did not answer the question (7/187; 4%). The most commonly reported criteria utilized to define treatment success was being seizure-free for 3 months or longer (133/187; 71%). Other criteria chosen included a 50% reduction in seizure frequency and/or decrease in severity of seizures (93/187; 50%), client satisfaction (67/187; 36%), lack of/minimal ASD side effects (62/187; 33%), and ASD serum concentration within the reference range (55/187; 29%).

For control of breakthrough seizures, most respondents recommended performing bloodwork and measuring ASD levels to optimize dose (114/187; 61%) and/or adding a new ASD (119/187; 64%). Other choices included replacing the current ASD with a new ASD (78/187; 42%) or increasing the dose of the existing drug without bloodwork or drug concentration (33/187; 18%). When asked about criteria for discontinuing ASDs, the most common reasons were toxicity/adverse effects (98/187; 52%) or being seizure-free for more than 6–12 months (98/187; 52%). Less commonly, respondents indicated that they do not recommend discontinuing for any reason (40/187; 21%) while none cited seizure freedom for 1–6 months.

#### 3.1.6 Attitudes and challenges related to IE management

**Figure 2:**
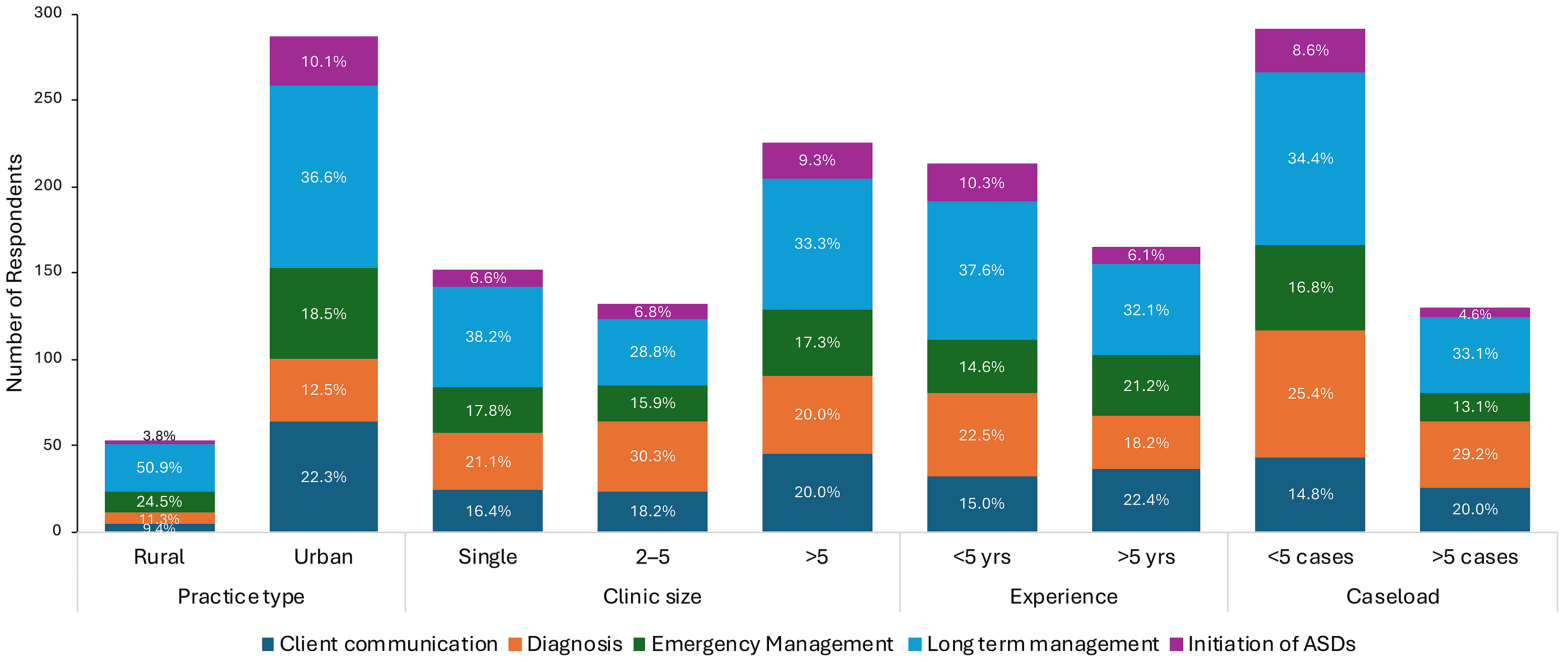
Stacked column chart illustrating which aspect of canine idiopathic epilepsy (IE) management the respondents found most challenging, stratified by practice type, experience, clinic size and case load. Values are displayed as percentages within each subgroup. ASD, anti-seizure drug.

The mean self-reported comfort level (ranging from 1/not comfortable to 5/very comfortable) in managing dogs with IE was 3.3 (SD 0.97). The distribution of ratings was: 1 (5/187; 3%), 2 (27/187; 14%), 3 (73/187; 39%), 4 (51/187; 27%), and 5 (23/187; 12%). The most challenging aspect of IE management was noted to be long-term management of seizures and side effects of ASDs (133/187; 71%). Other, less commonly chosen options included diagnosis (78/187; 42%), client communication regarding diagnosis and management (69/187; 37%), emergency seizure management (66/187; 35%), and initiation of therapy (32/187; 17%). Eight respondents (8/187; 4%) did not answer this question. A breakdown of the challenges by different demographic groups is shown in Figure 2.

Respondents sought information about canine IE from multiple sources, with advice from other primary care veterinarians rated the most used source by the most respondents (55/187; 29%). Other sources rated as most utilized by respondents included peer-reviewed literature (34/187; 18%), neurology books (34/187; 18%), post-graduate continuing education like seminars and conferences (23/187; 12%), and notes from veterinary school (17/187; 9%); no respondents ranked social media as their most used source of information. Nine respondents did not answer this question (9/187; 5%).

On a scale from 1 (not needed) to 5 (extremely beneficial) rating the value of access to continuing education information relating to the management of IE in dogs, the mean score was 4 (SD 0.84). The format for continuing education rated as most helpful by the most respondents was quick reference flow charts or treatment algorithms (82/187; 44%). Other responses included comprehensive information sheets or handouts (45/187; 24%), webinars or recordings (36/187; 19%), and access to published materials on the topic (24/187; 13%).

### 3.2 Phase II

**Figure 3:**
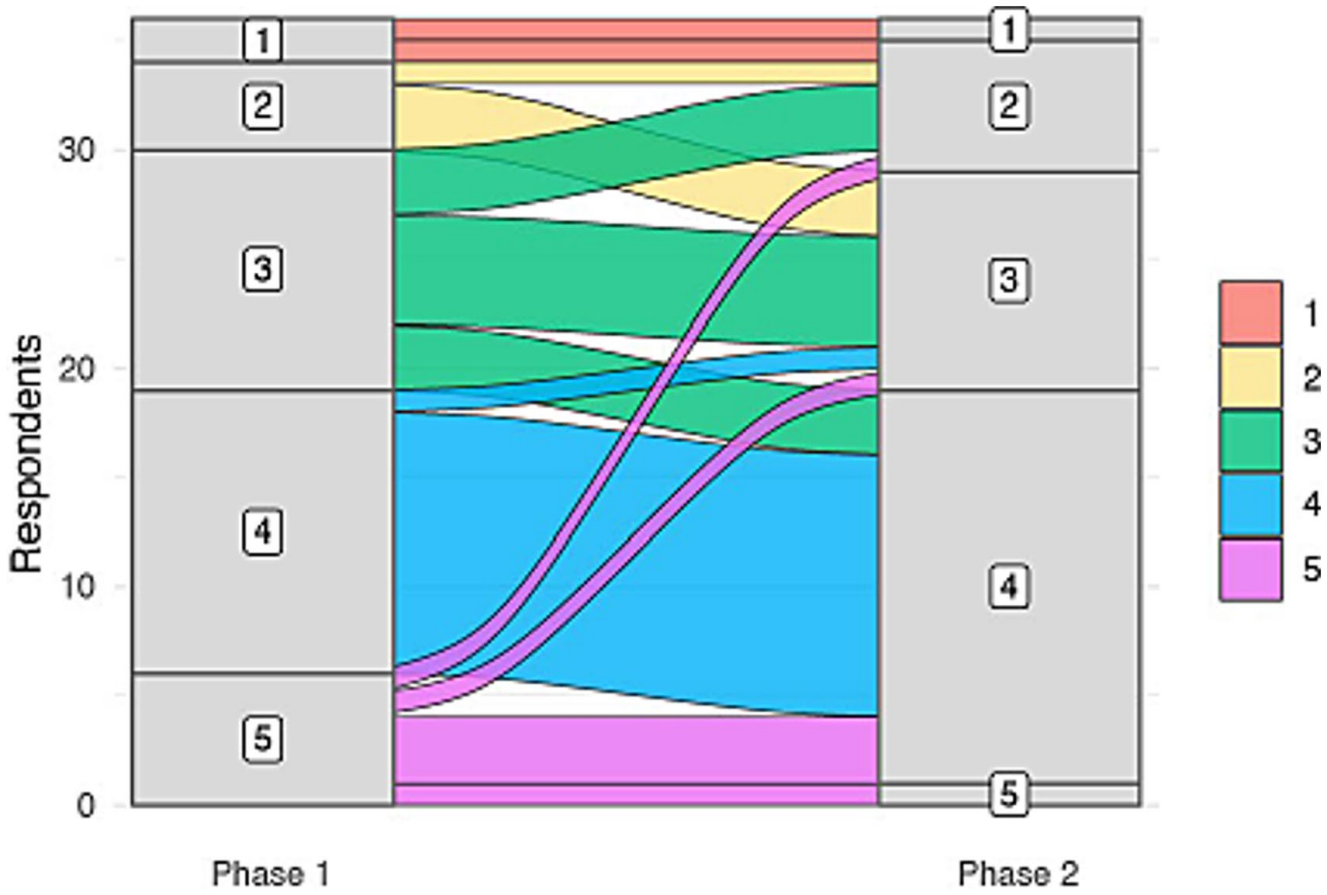
Alluvial plot illustrating changes in self-reported comfort levels in managing canine idiopathic epilepsy (IE) among primary care veterinarians in India between Phase I (*n* = 187) and Phase II (*n* = 36). Comfort levels were rated from 1 (very uncomfortable) to 5 (very comfortable). The width of each stratum represents the number of respondents at each comfort level within each phase, and the connecting flows depict transitions in individual responses between phases.

Thirty-six (36/187; 19%) participants from Phase I completed the Phase II questionnaire (Table 1). Phase II responders and non-responders were similar across clinic and clinician factors. Baseline (Phase I) mean comfort scores were also comparable between responders (3.47, SD 1.08) and non-responders (3.28, SD 0.96). In the 6 months between surveys, respondents managed 1–4 dogs with IE, with most managing 1–2 cases (30/36; 83%). Across questions relating to diagnosis, treatment, monitoring, medication adjustments and evaluating treatment success, the distributions of responses were qualitatively similar between Phase I and II. Phenobarbital remained the ASD prescribed most frequently, followed by levetiracetam. Long-term management of seizures and adverse effects of ASDs remained the most challenging aspects for clinicians. For Phase II, the mean self-reported comfort score for managing dogs with IE was 3.69 (SD 0.89). Most respondents (20/36; 56%) reported no change, with fewer reporting an increase (8/36; 22%) or decrease (8/36; 22%) in comfort level (Figure 3).

Most respondents reported referring to the educational materials 1–5 times per month (23/36; 64%). Ranking the educational materials from 1 (not beneficial) to 5 (extremely beneficial), most respondents chose 5 (17/36; 47%), followed by 4 (14/36; 39%) and 3 (5/36; 14%). Feedback received from Phase II participants regarding the educational materials included requests for adding flow charts, reading guides or references, current updates in diagnostic tools, and information on recent drug discoveries.

## 4 Discussion

This study provides initial insights into the current management practices and treatment decisions relating to canine IE in primary care practices across India. Among primary care veterinarians in India, phenobarbital was the most common ASD prescribed for IE. These results are comparable to other survey-based studies investigating management of canine IE by primary care veterinarians in the United Kingdom, Europe and Australia where approximately 88 to 99% of participants most frequently used phenobarbital for long term treatment (8, 9, 13). While studies from the United States on ASD use in dogs with suspected IE, encompassing both primary care providers and board-certified emergency and neurology specialists, also reported phenobarbital as the initial ASD of choice, this was selected by a smaller proportion, ranging from 35–66% of practitioners (10, 12). Our results provide further confirmation of the broad access and frequent clinical use of phenobarbital worldwide.

Levetiracetam was the second most utilized medication, more commonly as add-on than as the first medication. Levetiracetam was prescribed at a higher rate among practitioners in urban (vs rural) settings as well as those working at larger clinics, having more years of veterinary experience and managing a larger number of IE cases. Globally, levetiracetam administration varies widely, with this ASD typically recommended more frequently by neurology and emergency specialists (8–12). However, a recent study among US primary care practices showed levetiracetam was prescribed as the first drug almost as frequently as phenobarbital, which might reflect growing utilization in the non-specialist setting (12). In India, levetiracetam was approved for use in people in 2005 (22). This is in comparison to developed countries where it has been in use since the 1990s. The relatively newer introduction could have impacted regional availability of this medication and played a role in the lower reported frequency of its clinical utilization and greater preference by specific subsets of Indian veterinarians.

Respondents chose efficacy as the most common reason for selecting the initial and add-on ASD, accounting for nearly half and two thirds of responses, respectively. While we predicted that medication cost and availability would be major factors in decision-making among Indian veterinarians, cost was uncommonly selected and the impact of availability seemed to be true primarily among rural practitioners. Cost comparison of ASDs available in the Indian market demonstrated substantial price variation among different brands of the same medication, precluding direct comparisons (23). Despite this, levetiracetam demonstrated a higher price range (₹6.5–74.8 per tablet; approximately $0.08–$0.90 USD) compared to phenobarbital (₹0.6–2.2 per tablet; approximately $0.007–$0.03 USD). Other factors such as clinic size and clinician experience did not appear to have a major impact on reported reasoning for prescribing choices in our study. However, unidentified geographical, cultural, or educational influences might have contributed to our results as such factors have been suggested as a source variation in drug preferences in other studies (13). Additionally, few respondents reported managing dogs on multiple ASDs. This could highlight more limited experience using other ASDs apart from phenobarbital.

Most respondents recommended routine blood work and close to half of respondents recommended performing phenobarbital or potassium bromide blood concentration. However, 6% of respondents reported not routinely performing lab work for dogs on ASDs and about one third of respondents reported not routinely performing ASD levels. Across prior studies, the frequency of practitioners who report routinely monitoring serum concentrations of ASDs varies widely from 16–71% (8, 9, 11, 12). The most common reason cited by Japanese clinicians for not measuring ASD blood levels was they did not feel it was necessary, which likely reflects the frequent administration of zonisamide in Japan (11). In our study, the predominant reasons were financial restrictions and lack of access to these tests. Although the educational materials contained details on making changes to phenobarbital based on blood concentration, knowing that a reasonable proportion of practitioners are unable to perform these tests is an important factor. Given that prior consensus statements on the management of epilepsy recommend measuring ASD concentration when medication is initiated, dose changes are made, and/or seizure control is inadequate (4, 5) and testing availability will likely continue to expand in India, this highlights an important area for continuing education catered to this region.

The recognition of and response to various ASD adverse effects were variable and choices did not necessarily reflect anticipated medication effects or conform to published guidelines on IE management (7, 24–26). For example, while about one third of respondents used a decrease in liver function parameters as a marker for phenobarbital-induced hepatotoxicity, the most common choices related to ALP elevation, and just over 10% used any ALT elevation. Moreover, over 60% of respondents recommended tapering to discontinue phenobarbital when faced with sedation and ataxia after initiating treatment, adverse effects that published guidelines suggest should resolve in most cases within 1–2 weeks (5). Similarly, treatment success was largely defined by achieving a longer interval between seizures or decreased severity, but nearly half of respondents still reported a main goal with therapy was to achieve complete seizure freedom and just over half cited a lack of seizures for 6–12 months as a reason for ASD discontinuation. While such variability and discrepancies between management recommended by neurologists and implementation by primary care clinicians is commonly noted worldwide (8, 13), there could also be factors specific to developing countries. In the Indian veterinary curriculum, didactic instruction in the subject of neurology is limited and delivered by non-specialists. Additionally, the Veterinary Council of India does not have mandatory continuing education requirements for maintaining a veterinary license. This could result in fewer broadly accessible opportunities for post-graduate knowledge and skill development for Indian primary care practitioners. The results of our survey, which excluded clinicians with any structured training in veterinary neurology, highlights areas such as the veterinary curriculum or continuing education opportunities and specific topics that could be targeted for additional support to enhance clinical neurology knowledge among primary care practitioners in India.

Respondents found long term management of seizures and the side effects of ASDs to be the most challenging. This was the most selected response regardless of clinic type or size, caseload or number of years of experience in practice. The frequency of additionally selected challenges varied somewhat across practice or practitioner characteristics, but diagnosis of IE was the next most selected option by veterinarians with less than 5 years’ experience. This has been previously reported among newer veterinarians (8) and might reflect the lack of knowledge gained from more years of follow up care, especially since about two thirds of respondents treated fewer than 5 epileptic dogs per year. Additionally, access to advanced imaging is limited in India and might have contributed to diagnosis of IE being perceived as challenging. While this is likely to remain an obstacle in India and other developing countries, diagnosis based on an International Veterinary Epilepsy Taskforce tier 1 confidence level (age at onset, clinical examination and lab work) is reasonable in many dogs (4, 5). Therefore, educational resources could be tailored to prioritize making the diagnosis of IE when advanced imaging is not possible. Similarly, management guidelines could focus on needs that are independent of specific resources and advanced diagnostics such as client communication. Despite the perceived challenges, most respondents rated themselves reasonably comfortable in managing dogs with IE and scores were broadly comparable to those reported from a study out of the Netherlands (13). It is possible that our cohort captured veterinarians confident in managing straightforward cases for a relatively short duration but who recognized the potential for issues associated with managing complex cases or for a longer period of time.

The educational materials distributed in Phase II were developed to evaluate the feasibility and reception for this format of providing continuing educational support on the topic if IE in dogs. Importantly, they were distributed with instructions to use at their discretion and without a control group or explicit instructions that there would be a follow up assessment. While about two thirds of Phase II respondents reported referring to the materials 1–5 times per month and all rated them as beneficial, including nearly half ranking them as extremely beneficial, the median self-reported ratings of comfort with managing dogs with IE were similar between Phases I and II, and most respondents rated their comfort as the same. Similarly, most treatment and monitoring-related questions had similar distributions of the responses between Phases I and II. While some qualitative differences could reflect an impact of the educational materials, random variance and the very small sample size for Phase II limit conclusions that can be drawn about the intervention, beyond basic feasibility and positive reception. Additionally, the unchanged constraints of regional availability of resources are also important considerations.

To further evaluate the utility of the educational materials, it is possible that a longer interval between surveys might be important. Most respondents had only managed 1 or 2 epileptic dogs in the preceding 6 months, limiting the possibility of implementing recommendations outlined in the guidelines. The relatively high baseline confidence level may have limited the range of measurable improvement in Phase II and increased awareness of knowledge gaps following exposure to the materials may have contributed to unchanged or reduced confidence in some participants. It is also possible that the format or content was not ideal. The educational materials distributed in Phase II were concise and intended to allow for quick provision of information. This practical approach was supported by Phase I respondents rating quick reference flow charts or treatment algorithms as the most useful format for continuing education, but further optimization of content and delivery is likely needed. While explicit conclusions from this preliminary work are limited, since subjective clinician rated benefit was high and individual feedback regarding materials was positive, there appears to be value in further, more objectively exploring the usefulness of such materials in improving the management of IE. Future work could also investigate scalability for more participants and application to other developing countries and for other commonly managed chronic diseases.

Primary care veterinarians in the United Kingdom mostly seek information about canine IE from neurology specialists, followed by from other primary care veterinarians or peer reviewed literature (8). This is different from our study, where Indian veterinarians mostly sought information from other veterinarians and less commonly peer reviewed literature. This highlights a potential disparity in developing countries who lack specialists for consultation or referral and might have more limited access to published literature unless it is open access. While the perceived benefit of direct access to neurologists was not evaluated as part of our study, a future direction could be to develop strategies for remote but direct connection between specialists and primary care veterinarians. Various veterinary specialties have adopted different models of telemedicine in both developed and developing countries with positive feedback (27). The ability to discuss complex cases or concerns directly could provide a complementary means of continuing educational support.

Limitations of this study include small sample size and low response rate, common among survey-based studies. There are more than 80,000 veterinarians registered with the regulatory agency Veterinary Council of India and therefore, responses from the nearly 200 participants might not be broadly representative of small animal primary care veterinarians in India. A challenge faced during the Phase I survey period was filtering fraudulent responses. It is possible that some non-fraudulent responses were inadvertently excluded because of this filtering process, but we elected to remove any that could not be confirmed as valid responses from eligible individuals. It is possible that this process influenced our data set and introduced unintended attrition bias towards certain types of responders or response patterns. Additionally, data removal might have further reduced study numbers and impacted the validity and generalizability of results. Although the questionnaire was adapted from a previously validated survey, formal validation of the adapted version was not performed in the Indian veterinary setting. Consequently, its validity and reliability in this population cannot be assumed, and responses should be interpreted with this limitation in mind. While all Phase I respondents were offered participation in Phase II, the low retention rate for Phase II could have been due lack of willingness to spend time to undertake a survey or failure to understand the request for continued participation. The absence of a control group for Phase II is another limitation in evaluating efficacy of the educational intervention. The results of this work could be used to design future, larger studies that incorporate a control cohort to better investigate the practical value of the educational materials. This study also focused on dogs and veterinarians in India and results cannot necessarily be extrapolated to epilepsy management in other developing countries or species. Our questionnaire also did not include questions assessing management of emergency seizure scenarios, which was noted to be challenging by more than a third of our survey population and was recommended to be added to the educational materials.

## 5 Conclusion

This preliminary study identified challenges faced by Indian primary care veterinarians in the management of IE in dogs. Areas identified for further investigation and targeted continuing education support included various aspects of chronic case management such as therapeutic drug monitoring, ASD related adverse effects and their management, and assessing and communicating goals of treatment. While Phase II was preliminary and exploratory, the results showed a positive reception from Indian practitioners to pre-made educational materials developed in a quick reference format. Future studies could focus on tailoring the content and optimizing the format and delivery of educational materials to determine if they help to improve clinician confidence in chronic case management. Such initiatives might improve the regional standard of care for canine IE in India and possibly other developing countries and could be extrapolated to the management of other conditions in areas without specialist access.

## Data Availability

The raw data supporting the conclusions of this article will be made available by the authors, without undue reservation.
