# Supplementary material for: Management practices and continuing education in the treatment of canine idiopathic epilepsy among Indian primary care veterinarians
Source: Front Vet Sci. 2026 Jul 21;13:1868929. doi: 10.3389/fvets.2026.1868929 (PMC13433295; doi:10.3389/fvets.2026.1868929)
Supplement: Supplementary file 1 [file Data_Sheet_1.PDF]

Phase I Survey

Consent and Eligibility

Q1-3 relating to consent to participate were removed.

Q4 Are you a veterinarian in clinical practice who holds a license issued by the State Veterinary Council of India in the state where you practice?

- ◯ Yes
- ◯ No

Q5 Have you ever held any of the listed certifications/diplomas in veterinary neurology?

- ◯ ESAVS Certificate in Neurology
- ◯ Post graduate diploma in Veterinary Neurology from an accredited veterinary university
- ◯ ACVIM (Neurology) - American College of Veterinary Internal Medicine
- ◯ ECVN - European College of Veterinary Neurology
- ◯ I have not held any certificate in Veterinary Neurology
- ◯ Other (specify) ____

Q6 Have you managed at least two dogs with idiopathic epilepsy in the past 12 months?

- ◯ Yes
- ◯ No

Demographic and background

Q7 What is your age?

____

Q8 What is your gender?

- ◯ Male
- ◯ Female
- ◯ Non-binary / third gender
- ◯ Prefer not to say

Q9 How many years have you been working as a veterinarian in private practice?

- ◯ Less than 5 years
- ◯ 5 - 10 years
- ◯ 11 - 20 years
- ◯ More than 20 years

Q10 What is the setting of your current practice?

- ◯ Urban (City or town)
- ◯ Rural

Q11 How many veterinarians are currently employed in your clinic/hospital?

- ◯ I am the only veterinarian at my clinic / practice
- ◯ Less than 5
- ◯ 5-10
- ◯ 11-20
- ◯ More than 20

Diagnosis of Idiopathic Epilepsy and Initiation of Treatment

Q12 Which of the following criteria do you use to diagnose idiopathic epilepsy in dogs (Select all that apply)?

- ◯ Age of onset of seizures between 6 months to 6 years
- ◯ History of more than one generalized seizure
- ◯ Normal lab-work (including CBC, serum biochemistry and/or urinalysis)
- ◯ Normal hepatic function testing (ammonia +/- bile acids)
- ◯ Normal physical and neurologic examination between seizures
- ◯ Normal magnetic resonance imaging (MRI) of the brain and cerebrospinal fluid analysis (and/or infectious disease testing)

Q13 How do you interpret serum ammonia levels in dogs with seizures?

- ◯ Any elevation above reference range is considered the cause of the seizures
- ◯ Considered to be the cause of the seizures if elevated more than 2x of reference range
- ◯ I recommend pre and post prandial bile acids to confirm suspicion for hepatic dysfunction to confirm elevated ammonia

Q14 Approximately how many dogs with idiopathic epilepsy have you managed in the last 12 months?

- ◯ I have not managed a dog with idiopathic epilepsy in the last 12 months
- ◯ Less than 5
- ◯ 5-10
- ◯ 11-20
- ◯ More than 20

Q15 When do you recommend starting anti-seizure medication in dogs with idiopathic epilepsy? (Select all that apply)

- ◯ After the first seizure
- ◯ Multiple (2 or more) seizures in the preceding 3 -6 months
- ◯ 2 or more seizures in 24 h (cluster seizures)
- ◯ A seizure lasting > 5 min (status epilepticus)

Q16 What oral anti-seizure medications does your hospital stock currently? (Select all that apply)

- ◯ Levetiracetam (Keppra IR/XR, Levepsy, Levecetam)
- ◯ Phenobarbital (Gardenal, Luminal, Fenobarb)
- ◯ Potassium Bromide
- ◯ Zonisamide
- ◯ Diazepam (Valium)
- ◯ Other (specify) ____

Q17 Which of the following anti-seizure medications do you most commonly prescribe FIRST as long term treatment in idiopathic epilepsy in dogs? (Rank from 1 being most common to 4 being least common)
_______ Levetiracetam (Keppra, Levepsy, Levecetam)
_______ Phenobarbital (Gardenal, Luminal, Fenobarb)
_______ Potassium Bromide
_______ Zonisamide

Q18 What is the most common reason for choosing the FIRST anti seizure medication?

- ◯ Side effect profile
- ◯ Efficacy
- ◯ Availability in my area
- ◯ Cost
- ◯ Ease of administering the medication
- ◯ Other - specify ____

Q19 When using each of these anti-seizure medications as a sole agent, what is your starting dose (mg/kg) and frequency (q24h, q12h, q8h) for a dog?

- ◯ Phenobarbital ____
- ◯ Levetiracetam ____
- ◯ Zonisamide ____
- ◯ Potassium Bromide ____

Q20 When starting each of these anti-seizure medications, do you administer a loading dose?

- ◯ Yes, always
- ◯ Yes for phenobarbital or bromide
- ◯ Yes, but only in emergency seizure situations (cluster seizures or status epilepticus)
- ◯ No

Q21 Which of the following anti-seizure medications do you most commonly prescribe as an add-on long term medication for idiopathic epilepsy in dogs?

- ◯ Levetiracetam (Keppra, Levepsy, Levecetam)
- ◯ Phenobarbital (Gardenal, Luminal, Fenobarb)
- ◯ Potassium Bromide
- ◯ Zonisamide

Q22 What is the most common reason for choosing the add-on anti-seizure medication? (Select all that apply)

- ◯ Side effect profile
- ◯ Different route of metabolism than the first anti seizure medication
- ◯ Efficacy
- ◯ Cost
- ◯ Availability in my area
- ◯ Ease of administering the medication
- ◯ Other - specify ____

Q23 When using each of these anti-seizure drugs as an add-on agent, what is your starting dose (mg/kg) and frequency (q24h, q12h, q8h) for a dog?

- ◯ Phenobarbital ____
- ◯ Levetiracetam ____
- ◯ Zonisamide ____
- ◯ Potassium Bromide ____

Q24 What percentage of dogs with idiopathic epilepsy that you manage are on two or more long term anti-seizure medications?

- ◯ Less than 10%
- ◯ 10-50%
- ◯ 50-75%
- ◯ More than 75%

Q25 Do you recommend any other treatments for idiopathic epilepsy in dogs? (Select all that apply).

- ◯ Acupuncture
- ◯ Herbal Medicine (Please specify which product below)
  ____
- ◯ CBD supplement
- ◯ Physical rehabilitation/Laser therapy
- ◯ Diet or dietary supplementation (e.g., medium chain fatty acids/triglycerides)
- ◯ Other ____

Q26 Do you recommend any of the following dietary supplements for dogs with idiopathic epilepsy? (Select all that apply).

- ◯ Medium Chain Triglyceride (MCT) supplementation
- ◯ I do not make any specific diet recommendations
- ◯ Other (specify) ____

Monitoring

Q27 For dogs on phenobarbital or potassium bromide, when do you measure blood concentration of the drug: (Select all that apply).

- ◯ I do not measure blood concentration
- ◯ I do not prescribe either of these medications
- ◯ Performed after starting therapy
- ◯ Performed after making a dose change
- ◯ Performed if adverse effects occur
- ◯ Performed regularly (every 6-12 months)

Q28 If phenobarbital or potassium bromide drug blood levels are not regularly performed, why not? (Select all that apply)

- ◯ Pet owner financial restrictions
- ◯ Do not have access to these lab tests
- ◯ Do not recommend performing these tests
- ◯ Do not find these tests useful in seizure management
- ◯ Other reason ____

Q29 For dogs on any anti-seizure medication(s), when do you perform blood work (CBC and serum biochemistry)? (Select all that apply)

- ◯ Every 6-12 months
- ◯ Only performed if adverse effects occur
- ◯ Only performed if seizure control is poor
- ◯ Only performed for dogs on certain medications (specify which) ____
- ◯ Not routinely performed

Q30 When do you recommend discontinuing anti-seizure medications? (Select all that apply)

- ◯ I do not recommend discontinuing seizure medications for any reason
- ◯ If the dog is seizure free for 1-6 months
- ◯ If the dog is seizure free for 6-12 months or longer
- ◯ If the dog develops toxicity to medication/adverse effects

Q31 How do you approach the management of worsening seizure frequency despite the dog's anti-seizure treatment regimen? (Select all that apply)

- ◯ Make no change to medication(s) as intermittent seizures will occur
- ◯ Perform routine blood work and measure anti-seizure drug levels to optimize dose of existing drug(s)
- ◯ Addition of new anti-seizure drug
- ◯ Replace current anti-seizure drug with a new anti-seizure drug
- ◯ Increase dose of existing drug(s) without bloodwork or drug levels
- ◯ Other (specify which) ____

Q32 How do you manage common side effects of anti-seizure medication (sedation, lethargy, ataxia)? (Select all that apply)

- ◯ Discontinue the anti-seizure drug immediately
- ◯ Gradually taper dose and eventually discontinue
- ◯ Continue medication and monitor patient clinically for resolution of side effects
- ◯ Perform bloodwork and anti-seizure drug levels
- ◯ Recommend advanced imaging to look for other causes (Brain MRI)

Q33 What changes on routine bloodwork do you use to monitor for phenobarbital induced hepatotoxicity? (Select all that apply)

- ◯ Any ALP elevation
- ◯ Progressive ALP elevation on serial bloodwork (more than 2x high end of reference range)
- ◯ Any ALT elevation
- ◯ ALT elevation greater than ALP elevation or > 2x high end of reference range
- ◯ Decrease in liver function parameters (albumin, cholesterol, BUN, glucose)

Q34 What additional diagnostics do you recommend in cases of suspected phenobarbital induced hepatotoxicity? (Select all that apply)

- ◯ Phenobarbital serum level
- ◯ Serum ammonia
- ◯ Pre and post prandial bile acids
- ◯ Abdominal Ultrasound/CT
- ◯ Other (specify) ____
- ◯ None recommended

Q35 How do you treat phenobarbital induced hepatotoxicity?

- ◯ Gradually taper phenobarbital dose for eventual discontinuation
- ◯ Decrease the phenobarbital dose and monitor bloodwork and phenobarbital blood levels (every 2-3 weeks) for improvement
- ◯ Discontinue phenobarbital without tapering
- ◯ Initiate hepatoprotective treatment (Denamarin, Silybin) while continuing phenobarbital unchanged

Q36 How do you approach uncommon idiosyncratic side effects of phenobarbital (pancytopenia, necrolytic dermatitis, lymphadenopathy)?

- ◯ Gradually taper phenobarbital for eventual discontinuation
- ◯ Decrease phenobarbital dose and monitor for improvement
- ◯ Discontinue phenobarbital without tapering
- ◯ No change to phenobarbital dose or dosing

Q37 How do you approach bromism (bromide toxicity) characterized by excessive sedation, ataxia and tetraparesis? (Select all that apply)

- ◯ Measure bromide levels
- ◯ Hospitalise on IV fluids to facilitate renal excretion of bromide
- ◯ Discontinue potassium bromide
- ◯ Reduce potassium bromide dose
- ◯ No treatment pursued

Treatment Efficacy and Outcomes

Q38 What is your main goal when managing dogs with idiopathic epilepsy?

- ◯ Achieving complete seizure freedom
- ◯ Reducing seizure frequency and severity, but not complete freedom
- ◯ Minimizing side effects associated with anti-seizure drugs
- ◯ Minimizing pet-owners' financial burden associated with seizure management

Q39 What criteria do you use to determine whether anti-seizure treatment is successful? (Select all that apply).

- ◯ 50% decrease in seizure frequency and/or decreased severity of seizures
- ◯ Being seizure free for 3 months or longer
- ◯ Antiepileptic medication serum concentration within the reference range
- ◯ Client satisfaction
- ◯ Lack of or minimal drug related side effects
- ◯ Other ____

Q40 How comfortable do you feel managing dogs with Idiopathic Epilepsy currently? From 0 (very uncomfortable) to 5(very comfortable)

	0	1	2	3	4	5
Scale ()						

Q41 What aspect of managing idiopathic epilepsy do you find most challenging? (Select all that apply)

- ◯ Diagnosis
- ◯ When and how to initiate therapy for seizures
- ◯ Long term management of seizures and side effects of anti-seizure medications
- ◯ Emergency seizure management
- ◯ Client Communication about the diagnosis or management of their dog's epilepsy
- ◯ Other ____

Q42 Where do you most commonly seek information about idiopathic epilepsy treatment? (Rank from most used to least used)
_______ Advice from other primary care veterinarians
_______ Materials/notes retrained from veterinary college education
_______ Post-graduate continuing education (seminars, conferences)
_______ Advice from technical advisors of relevant pharmaceutical companies
_______ Internet searches
_______ Neurology textbooks
_______ Peer-reviewed published veterinary literature (primary or review journal articles)
_______ Social media

Q43 On a scale from 1 (not beneficial) to 5 (extremely beneficial) how beneficial would it be to have access to continuing education materials relating to the management of idiopathic epilepsy in dogs?

	1	2	3	4	5
Scale ()	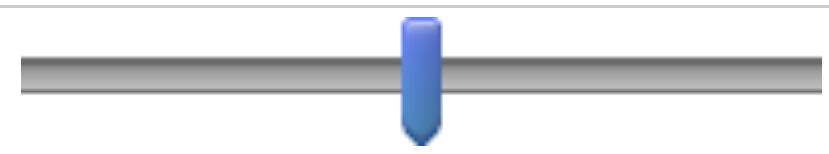				

Q44 In what format would this information be the most clinically applicable to you? (Rank from most helpful to least)
_______ Comprehensive information sheets/handouts
_______ Quick reference flow charts or treatment algorithms
_______ Continuing education webinars or recordings
_______ Access to published materials on the topic (consensus statements and other peer-reviewed publications)
