# Supplementary material for: Management practices and continuing education in the treatment of canine idiopathic epilepsy among Indian primary care veterinarians
Source: Front Vet Sci. 2026 Jul 21;13:1868929. doi: 10.3389/fvets.2026.1868929 (PMC13433295; doi:10.3389/fvets.2026.1868929)
Supplement: Supplementary file 2 [file Data_Sheet_2.PDF]

Phase II Survey

Consent and Eligibility

Q1-3 relating to consent to participate were removed.

Demographic and background

Q4 How many Idiopathic Epilepsy dogs have you managed in the last 6 months?

- ○ 1-5
- ○ 6-10
- ○ 11-15
- ○ 16-20

Q5 Has your area of employment changed since the previous survey?

- ○ Yes
- ○ No

Q6 If yes, then what is the setting of your current practice?

- ○ Urban
- ○ Rural

Diagnosis of Idiopathic Epilepsy and Initiation of Treatment

Q7 Which of the following criteria do you use to diagnose idiopathic epilepsy in dogs (Select all that apply)?

- ○ Age of onset of seizures between 6 months to 6 years
- ○ History of more than one generalized seizure
- ○ Normal lab-work (including CBC, serum biochemistry and/or urinalysis)
- ○ Normal hepatic function testing (ammonia +/- bile acids)
- ○ Normal physical and neurologic examination between seizures
- ○ Normal magnetic resonance imaging (MRI) of the brain and cerebrospinal fluid analysis (and/or infectious disease testing)

Q8 How do you measure and interpret elevated serum ammonia levels?

- ◯ Test is performed within 15 minutes of blood draw and stored in cold environment
- ◯ No specific extra precautions for blood sample handling and storage
- ◯ Any elevation above reference range is considered significant
- ◯ Considered significant if elevated more than 2x of reference range
- ◯ I perform pre and post prandial bile acids to confirm suspicion for hepatic dysfunction

Q9 When do you recommend starting anti-seizure medication in dogs with idiopathic epilepsy? (Select all that apply)

- ◯ After the first seizure
- ◯ Multiple (2 or more) seizures in the preceding 3-6 months
- ◯ 2 or more seizures in 24h (cluster seizures)
- ◯ A seizure lasting > 5 min (status epilepticus)

Q10 Which of the following anti-seizure medications do you most commonly prescribe FIRST as long term treatment in idiopathic epilepsy in dogs? (Rank from 1 being most common to 4 being least common)

_______ Levetiracetam (Keppra, Levepsy, Levecetam)
_______ Phenobarbital (Gardenal, Luminal, Fenobarb)
_______ Potassium Bromide
_______ Zonisamide

Q11 What is the most common reason for choosing the FIRST anti-seizure medication?

- ◯ Side effect profile
- ◯ Efficacy
- ◯ Availability in my area
- ◯ Cost
- ◯ Ease of administering the medication
- ◯ Other - specify ____

Q12 When using each of these anti-seizure medications as a sole agent, what is your starting dose (mg/kg) and frequency (q24h, q12h, q8h) for a dog?

- ◯ Phenobarbital ____
- ◯ Levetiracetam ____
- ◯ Zonisamide ____
- ◯ Potassium Bromide ____

Q13 When starting each of these anti-seizure medications, do you administer a loading dose?

- ◯ Yes, always
- ◯ Yes for phenobarbital or bromide
- ◯ Yes, but only in emergency seizure situations (cluster or status epilepticus)
- ◯ No

Q14 Which of the following anti-seizure medications do you most commonly prescribe as an add-on long term medication for idiopathic epilepsy in dogs?

- ◯ Levetiracetam (Keppra, Levepsy, Levecetam)
- ◯ Phenobarbital (Gardenal, Luminal, Fenobarb)
- ◯ Potassium Bromide
- ◯ Zonisamide

Q15 What is the most common reason for choosing the add-on anti-seizure medication? (Select all that apply)

- ◯ Side effect profile
- ◯ Different route of metabolism than the first anti-seizure medication
- ◯ Efficacy
- ◯ Cost
- ◯ Availability in my area
- ◯ Ease of administering the medication
- ◯ Other - specify ____

Q16 When using each of these anti-seizure drugs as an add-on agent, what is your starting dose (mg/kg) and frequency (q24h, q12h, q8h) for a dog?

- ◯ Phenobarbital____
- ◯ Levetiracetam ____
- ◯ Zonisamide ____
- ◯ Potassium Bromide ____

Q17 Do you recommend any other treatments for idiopathic epilepsy in dogs? (Select all that apply).

- ◯ Acupuncture
- ◯ Herbal Medicine (Please specify which product below)
  ____
- ◯ CBD supplement
- ◯ Physical therapy/Laser therapy
- ◯ Diet or dietary supplementation (e.g., medium chain fatty acids/triglycerides)
- ◯ Other ____

Q18 Do you recommend any of the following specific diets/dietary supplements for dogs with idiopathic epilepsy? (Select all that apply).

- ◯ Medium Chain Triglyceride (MCT) supplementation
- ◯ I do not make any specific diet recommendations
- ◯ Other (specify) ____

Monitoring

Q19 For dogs on phenobarbital or potassium bromide, when do you measure blood concentration of the drug: (Select all that apply).

◯ I do not measure blood concentration

◯ I do not prescribe either of these medications

◯ Performed after starting therapy

◯ Performed after making a dose change

◯ Performed if adverse effects occur

◯ Performed regularly (every 6-12 months)

Q20 If phenobarbital or potassium bromide drug blood levels are not regularly performed, why not? (Select all that apply)

◯ Pet owner financial restrictions

◯ Do not have access to these lab tests

◯ Do not recommend performing these tests

◯ Do not find these tests useful in seizure management

◯ Other reason ____

Q21 For dogs on any anti seizure medication(s), when do you perform blood work (CBC and serum biochemistry)? (Select all that apply)

◯ Every 6-12 months

◯ Only performed if adverse effects occur

◯ Only performed if seizure control is poor

◯ Only performed for dogs on certain medications (specify which) ____

◯ Not routinely performed

Q22 When do you recommend discontinuing anti-seizure medications? (Select all that apply)

- ◯ I do not recommend discontinuing seizure medications for any reason
- ◯ If the dog is seizure free for 1-3 months
- ◯ If the dog is seizure free for 6-12 months or longer
- ◯ If the dog develops toxicity to medication/adverse effects

Q23 How do you approach the management of worsening seizure frequency despite the dog's anti-seizure treatment regimen? (Select all that apply)

- ◯ Make no change to medication(s) as intermittent seizures will occur
- ◯ Perform routine blood work and measure anti-seizure drug levels to optimize dose of existing drug(s)
- ◯ Addition of new anti-seizure drug without any bloodwork
- ◯ Replace current anti-seizure drug with a new anti-seizure drug
- ◯ Increase dose of existing drug(s) without bloodwork or drug levels
- ◯ Other (specify which) ____

Q24 How do you manage common side effects of anti-seizure medication (sedation, lethargy, ataxia)? (Select all that apply)

- ◯ Discontinue the anti-seizure drug immediately
- ◯ Gradually taper dose and eventually discontinue
- ◯ Continue medication and monitor patient clinically for resolution of side effects
- ◯ Perform bloodwork and anti-seizure drug levels
- ◯ Recommend advanced imaging to look for other causes (Brain MRI)

Q25 What changes on routine bloodwork do you use to monitor for phenobarbital induced hepatotoxicity? (Select all that apply)

- ○ Any ALP elevation
- ○ Progressive ALP elevation on serial bloodwork (more than 2x high end of reference range)
- ○ Any ALT elevation
- ○ ALT elevation greater than ALP elevation (or > 2x high end of reference range)
- ○ Decrease in liver function parameters (albumin, cholesterol, BUN, glucose)

Q26 What additional diagnostics do you recommend in cases of suspected phenobarbital induced hepatotoxicity? (Select all that apply)

- ○ Phenobarbital serum level
- ○ Serum ammonia
- ○ Pre and post prandial bile acids
- ○ Abdominal Ultrasound/CT
- ○ Other (specify) ____
- ○ None recommended

Q27 How do you treat phenobarbital induced hepatotoxicity?

- ○ Gradually taper phenobarbital dose for eventual discontinuation
- ○ Incrementally decrease the phenobarbital dose and monitor bloodwork and phenobarbital blood levels (every 2-3 weeks) for improvement
- ○ Discontinue phenobarbital without tapering
- ○ Initiate hepatoprotective treatment (Denamarin, Silybin) while continuing phenobarbital unchanged

Q28 How do you approach uncommon idiosyncratic side effects of phenobarbital (pancytopenia, necrolytic dermatitis, lymphadenopathy)?

- ◯ Gradually taper phenobarbital for eventual discontinuation
- ◯ Discontinue phenobarbital without tapering
- ◯ Start on a new anti-seizure drug and continue on phenobarbital
- ◯ No change to phenobarbital dose or dosing

Q29 How do you approach bromism (bromide toxicity) characterized by excessive sedation, ataxia and tetraparesis? (Select all that apply)

- ◯ Measure bromide levels
- ◯ Hospitalise on IV fluids to facilitate renal excretion of bromide
- ◯ Discontinue potassium bromide
- ◯ Reduce potassium bromide dose
- ◯ No treatment pursued

Treatment Efficacy and Outcomes

Q30 What is your main goal when managing dogs with idiopathic epilepsy?

- ◯ Achieving complete seizure freedom
- ◯ Reducing seizure frequency and severity, but not complete freedom
- ◯ Minimizing side effects associated with anti-seizure drugs
- ◯ Minimizing pet-owners' financial burden associated with seizure management

Q31 What criteria do you use to determine whether anti-seizure treatment is successful? (Select all that apply).

- ◯ 50% decrease in seizure frequency and/or decreased severity of seizures
- ◯ Being seizure free for 3 months or longer
- ◯ Antiepileptic medication serum concentration within the reference range
- ◯ Client satisfaction
- ◯ Lack of or minimal drug related side effects
- ◯ Other ____

Q32 How comfortable do you feel managing dogs with Idiopathic Epilepsy currently? From 0 (very uncomfortable) to 5(very comfortable)

	0	1	2	3	4	5
Scale ()						

Q33 What aspect of managing idiopathic epilepsy do you find most challenging? (Select all that apply)

- ◯ Diagnosis
- ◯ When and how to initiate therapy for seizures
- ◯ Long term management of seizures and side effects of anti-seizure medications
- ◯ Emergency seizure management
- ◯ Client Communication about the diagnosis or management of their dog’s epilepsy
- ◯ Other ____

Q34 How often did you refer to the educational materials in the last 6 months?

- ◯ Less than one time per month
- ◯ 1-5 times per month
- ◯ 6-10 times per month
- ◯ More than 10 times per month

Q35 On a scale from 1 (not beneficial) to 5 (extremely beneficial) how beneficial were the provided continuing education materials relating to the management of idiopathic epilepsy in dogs?

	1	2	3	4	5
Scale ()					

Q36 Please provide any feedback regarding the educational materials to guide further studies.
