# Supplementary material for: Management practices and continuing education in the treatment of canine idiopathic epilepsy among Indian primary care veterinarians
Source: Front Vet Sci. 2026 Jul 21;13:1868929. doi: 10.3389/fvets.2026.1868929 (PMC13433295; doi:10.3389/fvets.2026.1868929)
Supplement: Supplementary file 3 [file Data_Sheet_3.PDF]

STARTING MAINTENANCE ANTI-SEIZURE MEDICATION

HOW to treat?

1. Establish dog's general health (physical exam +/- baseline CBC, serum biochemistry)
2. Choose drug to start with first:

	**Phenobarbital**	**Levetiracetam**	**Potassium bromide**	**Zonisamide**
**Starting dose:**	2-3mg/kg PO q12h	20-30mg/kg PO q8h (30-35mg/kg PO q12 for XR*)	30-40mg/kg PO q24h (or divided into q12h dosing)	5-10mg/kg PO q12h (8-10mg/kg if also on phenobarbital)
**Pros:**	Most effective!	Very safe!	Effective!	Safe!
**Limitations**	More side effects & monitoring; avoid in liver dysfunction	Less effective (than pheno); 3x/day administration (unless XR)	More side effects; challenging to titrate dose	Less info on efficacy; avoid in liver dysfunction
**Initial serum level?**	2-3 weeks after starting	Not necessary	3-4 months after starting	Not necessary, consider if also on phenobarbital

*XR = levetiracetam extended-release formulation

LOADING MAINTENANCE ANTI-SEIZURE MEDICATION

WHEN to 'LOAD'?

1. Dogs presented in Status Epilepticus or with cluster seizures
2. Load *in addition to* emergency treatment with benzodiazepines
3. Goal: Reach targeted drug level more quickly, then continue maintenance therapy

HOW to 'LOAD'?

	Phenobarbital	Levetiracetam	Potassium bromide
**Loading dose**	16-20mg/kg	60mg/kg	500mg/kg
**Route**	IV >> PO	IV >> PO	PO
**Administration protocol**	• 4mg/kg q4-6hrs (most common), speed up dosing interval if needed<br>• 16mg/kg once (less common)	• 60mg/kg once	• 100mg/kg/day (or 50mg/kg q12h) x 5 days<br>• If needed, speed up dosing interval: 100mg/kg q6h for 4-5 doses
**Considerations**	Sedation & ataxia (especially if giving as one large dose)	Sedation & ataxia (usually mild)	Sedation & ataxia

DETERMINING ANTI-SEIZURE MEDICATION "SUCCESS"

GOAL for treating IE: Optimize quality of life by decreasing seizures while minimizing medication side effects

Outcome Target	Measures of 'success' after starting (or adjusting) anti-seizure medication:	Communication with owners
**Seizure Frequency**	-Reduced by ≥50%<br>OR<br>-Interval between seizures ≥3x interval prior to starting medication<br>OR<br>-One seizure no more than every 1-3 months	-Complete seizure freedom is uncommon/rare<br>->1yr seizure-free before consider weaning off meds<br>-25-30% of dogs have 'refractory IE' = inadequate control despite ≥2 anti-seizure medications<br>-Control can wax and wane<br>-IE can progress over time
**Seizure Severity**	-No further episodes of status epilepticus<br>OR<br>-Occasional clusters of ≤3 seizures not requiring hospitalization<br>OR<br>-Shorter or less severe seizures or post-ictal phase	-In dogs prone to cluster seizures, eliminating clusters is uncommon/rare<br>-Control can wax and wane<br>-IE can progress over time
**Medication side effects**	-Sedation and ataxia mild or absent<br>AND<br>-Other drug-specific side effects (e.g., PU/PD for phenobarbital) manageable<br>AND<br>-No signs of drug-specific toxicity (e.g., phenobarbital-induced liver toxicity or bromism)	-Most anti-seizure medications have some side effects<br><br>-Balance with severity of epilepsy

NC STATE College of Veterinary Medicine

ADJUSTING MAINTENANCE ANTI-SEIZURE MEDICATION IN DOGS WITH *POOR SEIZURE CONTROL*

Step 1: Confirm poor control (see: 'starting medication' and 'determining success' handouts for guidelines)
Step 2: Confirm owner administration compliance
Step 3: Optimize current medication prior to addition of a second (or third…) medication

	**Phenobarbital**	**Levetiracetam**	**Potassium bromide**	**Zonisamide**
**Lab work**	phenobarbital level +/- chemistry panel	+/- levetiracetam level	bromide level	+/- zonisamide level
**Ceiling dose or serum level**	Targeted level:<br>20-30 umol/L,<br>up to 35 umol/L if more severe epileptic	Ceiling dose:<br>≥60mg/kg q8h<br>(or ≥90mg/kg q12h)<br>-above this no further efficacy likely	Targeted level:<br>1-2 mg/mL (100-200 mg/dL) (if also on pheno)<br>2-3 mg/mL (200-300 mg/dL) (if not on pheno)	Ceiling dose:<br>≥15-20mg/kg q12h<br>-above this, no further efficacy likely<br>Targeted level: 10-40 mcg/ml
**'Rules' for change: dose increase**	-If room to increase based on level (and side effects), calculate targeted new dose:<br>$\frac{\text{current level}}{\text{current dose(mg)}} = \frac{\text{target level}}{\text{X (mg)}}$<br>solve for 'X,' pick the closest tablet size(s)	-Increase dose to effect (25-33% increments)	-If room to increase based on level (and side effects), calculate targeted new dose:<br>(2.5mg/ml – bromide level) x 20 = mg/kg/d to add to current dose<br>OR<br>(250mg/dl – bromide level) x 0.2 = mg/kg/d to add to current dose<br>*use 2.0 or 200mg/dL if also on pheno	-Increase dose to effect (25-33% increments)<br>+/- check level 1-2 weeks after dose change

	-Obtain level 2-3 weeks after dose change		OR<br>Increase dose in 10-33% increments<br>- If rapid increase desired, partially load (100mg/kg/d x 2-3d), then start new maintenance dose<br>- Obtain level 3-4 months after dose change	
**'Rules' for change: if control inadequate but dose increase not possible**	-If level close to 30umol/L, add/increase other medication<br><br>- If level >35umol/L, use formula to decrease dose to target 20-30umol/L + add/increase other medication	- If already ≥60mg/kg q8h, add/increase other medication	-If level >3mg/ml and intolerable side effects, dose reduce by ~20-25% + add/increase other medication	- If already ≥20mg/kg q12h, add/increase other medication

MONITORING DOGS ON PHENOBARBITAL

When	What Tests	Interpretation	Recommendations
**2-3 weeks after starting or any dose change**	phenobarbital level<br><br>+/- CBC, chemistry panel (after starting or if >6 months since last check)	a) If blood work shows no significant abnormalities, level is in targeted range and dog is doing well: no changes<br>b) If anemia, leukopenia and/or thrombocytopenia: suspect idiosyncratic reaction (typically develops <1year into therapy)	a) Routine rechecks of blood work and phenobarbital level every 6-12 months<br>b) Taper rapidly (1-2 days) and discontinue*
**Routine checks** (every 6-12 months lifelong)	CBC, chemistry panel<br><br>phenobarbital level	c) If seizure control is adequate, side effects are acceptable, level is in targeted range, and lab work is unremarkable: no changes<br>d) If elevated ALP alone with targeted level: suspect induction from phenobarbital treatment, no changes<br>e) If ALT very elevated and >> ALP: suspect liver abnormality	c) Continue with routine rechecks of blood work and phenobarbital level every 6-12 months<br>d) Same as ‘c’<br>e) Consider abdominal ultrasound, +/- ammonia or bile acids; possible taper or discontinue

		f) If low albumin (or progressively declining on serial lab work) +/- low cholesterol, BUN, or blood glucose: suspect liver dysfunction<br>g) If elevated phenobarbital level (>35umol/L) with no signs of liver dysfunction on chemistry panel<br>h) If elevated phenobarbital level (>35umol/L) WITH signs of liver dysfunction on chemistry panel	f) Ammonia or bile acids; if mild liver dysfunction: dose reduce + recheck lab work and level, **OR** if severe: taper rapidly + discontinue*<br>g) Reduce dose and recheck level<br>h) Same as ‘f’
**Persistently sedate or ataxic**	CBC, chemistry panel<br><br>phenobarbital level	i) Sensitive dog or level too high	i) Reduce dose, recheck level and monitor for improvement in signs
**Sick dog: lethargy, weakness, ataxia, anorexia, vomiting, diarrhea, etc.**	CBC, chemistry panel, urinalysis, phenobarbital level<br><br>+/- pre and post prandial bile acids	j) If targeted phenobarbital level, but other abnormalities identified (on physical exam and/or routine lab work): suspect other disease<br>k) If icteric + signs of liver failure<br>l) If cytopenias: suspect idiosyncratic reaction	j) Proceed with systemic investigation<br>k) Taper rapidly (1-2 days) and discontinue*<br>l) Taper rapidly (1-2 days) and discontinue*

*Start/load or increase other anti-seizure medication (avoid zonisamide if phenobarbital-induced liver toxicity).

MONITORING DOGS ON LEVETIRACETAM

When	What	Interpretation	Treatment and Follow-up
Routine checks (every 12 months lifelong)	CBC, chemistry panel	No lab work abnormalities and doing well: no changes	Routine rechecks and blood work every 12 months
Persistent sedation, ataxia or behavior changes (uncommon)	CBC, chemistry panel	a) No lab work abnormalities: sensitive dog<br>b) Severe renal dysfunction: difficulty excreting<br>c) If no improvement after dose reduction: sensitive dog<br>d) If also on other anti-seizure medications: consider their contribution to signs	a) Decrease dose by 20-25%* and monitor for improvement in signs<br>b) Same as 'a' or change medication*<br>c) Further dose reduction or taper and discontinue (2-3 weeks)*<br>d) Refer to drug-specific monitoring information

*Start/increase other anti-seizure medication, depending on control

NC STATE College of Veterinary Medicine

MONITORING DOGS ON POTASSIUM BROMIDE (KBr)

When	What	Interpretation	Recommendations
**Routine checks (every 12 months lifelong)**	CBC, chemistry panel<br><br>bromide level	If blood work is unremarkable (ignore: high chloride), level is within targeted reference range, seizure control is adequate, side effects are acceptable: no changes	Continue routine lab work and bromide level every 12 months<br><br>*Important:* give with food & maintain a consistent diet while on bromide!
**Persistent sedation/ ataxia**	CBC, chemistry panel<br>bromide level<br><br>+/- phenobarbital level (if also on pheno)	a) If level >3mg/ml (or >300mg/dL): level high, suspect bromism<br>b) If bromide level 2-3 mg/ml (or 200-300mg/dL) with therapeutic pheno level: suspect sensitive dog	a) Reduce dose (25-33%), monitor for improvement, recheck level in 3-4 months*<br>b) Reduce KBr dose (10-33%), no change to pheno, monitor for improvement, recheck level in 3-4 months*
**Vomiting, diarrhea**	CBC, chemistry panel<br>bromide level<br>+/- abdominal ultrasound, pancreatic lipase immunoreactivity (cPLI)	a) Mild blood work changes: gastric irritation from bromide<br>b) Increased WBC, elevated liver enzymes, increased cPLI: bromide-induced pancreatitis or gastroenteritis	a) Give with food, divide into twice daily dosing<br>b) If mild or first episode: supportive care + no change to medication OR if severe or recurrent GI signs: discontinue* (no taper required due to slow metabolism)

*Start or increase other anti-seizure medication, depending on control

MONITORING DOGS ON ZONISAMIDE

When	What	Interpretation	Recommendations
**Routine checks** (every 12 months lifelong)	CBC, chemistry panel	If no lab work abnormalities and doing well: no changes	Routine rechecks and blood work every 12 months
**Persistent sedation, ataxia, inappetence**	CBC, chemistry panel<br><br>zonisamide level	a) If lab work normal, zonisamide level within targeted range: sensitive dog<br>b) If lab work normal, zonisamide level >40mcg/ml: level too high	a) Reduce dose (20-25%), monitor for improvement, recheck levels in 1-2 weeks<br>b) Same as ‘a’
**New-onset sedation, ataxia** *after combining with phenobarbital*	CBC, chemistry panel zonisamide level + phenobarbital level	c) If zonisamide level >40mcg/ml: level too high<br>d) If phenobarbital level >35umol/L: level too high	c) Same as ‘a’<br>d) Reduce dose (see: adjusting medications), recheck level 2-3 weeks
**Sick dog: vomiting, diarrhea, icterus, inappetence** (*weeks to several months after starting*)	CBC, chemistry panel, +/- bile acids or ammonia, abdominal ultrasound	Markedly elevated ALT, ALP, GGT, total bilirubin +/- bile acids/ammonia:<br>suspect idiosyncratic zonisamide-induced hepatotoxicity	Taper rapidly (1-2 days) and discontinue*

*Start/increase other anti-seizure medication, depending on control (avoid phenobarbital with hepatotoxicity)

NC STATE College of Veterinary Medicine
